# Treatment Adherence and Outcomes in Patients with Tuberculosis Treated with Telemedicine: A Scoping Review

**DOI:** 10.3390/tropicalmed10030078

**Published:** 2025-03-17

**Authors:** Kikelomo Sabainah Olowoyo, Deborah Tolulope Esan, Paul Olowoyo, Babatunji Emmanuel Oyinloye, Israel Opeyemi Fawole, Segun Aderibigbe, Mary Opeyemi Adigun, David Bamidele Olawade, Theophilus Olaide Esan, Benedict Tolulope Adeyanju

**Affiliations:** 1 Department of Medine, Ekiti State University Teaching Hospital, Ado-Ekiti 362103, Nigeria; olowoyoks80@gmail.com; 2 Faculty of Nursing Sciences, Bowen University, Iwo 232102, Nigeria; 3 Department of Medicine, Federal Teaching Hospital Ido-Ekiti, Afe Babalola University, Ado-Ekiti 360211, Nigeria; paulolowoyo@gmail.com; 4 Institute for Drug Research and Development, S.E. Bogoro Center, Afe Babalola University, Ado-Ekiti 360211, Nigeria; babatunjioe@abuad.edu.ng; 5 Phytomedicine, Biochemical Toxicology and Biotechnology Research Laboratories, Department of Biochemistry, College of Sciences, Afe Babalola University, Ado-Ekiti 360211, Nigeria; 6 Biotechnology and Structural Biology (BSB) Group, Department of Biochemistry and Microbiology, University of Zululand, Kwa-Dlangezwa 3886, South Africa; 7 Institute of Nursing Research, Osogbo 232111, Nigeria; fawoleisrael@gmail.com; 8 Department of Surgery, Federal Teaching Hospital Ido-Ekiti, Ido Ekiti 371101, Nigeria; addribigbe20042003@yahoo.com; 9 Department of Adult Health/Mental Health Nursing, University of Medical Sciences Ondo, Ondo 351104, Nigeria; maryopeyemi16@gmail.com; 10 Department of Allied and Public Health, School of Health, Sport and Bioscience, University of East London, London E16 2RD, UK; 11 Department of Ear Nose and Throat, Federal Teaching Hospital Ido-Ekiti, Ido Ekiti 371101, Nigeria; esant.olaide@gmail.com; 12 Department of Obstetrics and Gynaecology, Afe Babalola University, Ado-Ekiti 360211, Nigeria; adeyanjubt@abuad.edu.ng

**Keywords:** treatment adherence, outcomes, pulmonary tuberculosis, telemedicine, scoping review

## Abstract

Patient non-adherence to drug usage is a major barrier to treating tuberculosis (TB). Telemedicine has shown promise in treatment monitoring and evaluation. This paper aims to explore scientific evidence of telemedicine application in TB treatment to promote widespread adoption in areas that are remote or have poor road networks from health facilities. Articles published from 2010 to 2023 on the adherence and outcomes in pulmonary tuberculosis with the use of telemedicine were reviewed. A scoping review of the studies was conducted by two authors independently, following the PRISMA guidelines to identify relevant articles. Telemedicine interventions have shown improvements in medication adherence, treatment completion, cure rates, and smear conversion among TB patients. The available evidence supports the beneficial effect of telemedicine in improving treatment adherence and outcomes in patients with pulmonary tuberculosis. However, the effect and outcomes varied across studies, indicating the need for further research and standardization of telemedicine interventions.

## 1. Introduction

Globally, tuberculosis (TB) is a major communicable disease affecting millions of persons, and according to the “Global Tuberculosis Report 2024”, there were 10.8 million new TB cases globally in 2023, with an incidence of 134 per 100,000 population [1]. A combination of antibiotics is used in treating TB over the course of a minimum period of 6 to 9 months. Directly observed treatment short-course (DOTS) has been the standard treatment protocol, which involves the supervised administration of medications to ensure adherence [1]. However, this method, though strong among the other interventional strategies, has not proved efficient in the control of TB with the emergence of drug-resistant tuberculosis, including extensively drug-resistant (XDR) and multidrug-resistant (MDR) strains [2].

Various strategies have been implemented globally to combat the spread of tuberculosis. WHO’s End TB Strategy seeks to reduce TB fatalities, incidence, and out-of-pocket expenses associated with TB care by 2035 [3]. The strategy emphasizes early diagnosis and treatment, expanding access to quality care, and confronting the social determinants of tuberculosis. Treatment adherence is a major obstacle to TB management because patients typically find the 6-month medication regimen too onerous, resulting in either delayed treatment beginning, treatment interruption, or treatment discontinuation (treatment default) [4]. Each year, non-adherence increases the number of infectious days, heightens the risk of relapse, and contributes to the development of drug-resistant tuberculosis [5].

Significant implementation obstacles exist for conventional directly observed therapy [4]. Adherence incurs ongoing non-monetary costs for patients and their families, even when TB medications themselves are free [6]. These costs include physical side effects, lost productivity, transportation expenses, social stigma, and a possible affront to patients’ autonomy [7]. One enduring challenge is the fact that supervision and assistance often must be provided face-to-face; this is especially problematic in low-resource areas, where patients may be geographically dispersed and difficult to access.

Numerous adherence-enhancement solutions based on DOT have been explored to solve these issues. Reminder systems, defaulter action, education, and peer support are all examples of interventions that try to improve adherence but do not address the underlying motivation issue, with varying degrees of success [8,9]. However, studies of monetary incentives reveal that patients who get material benefits are much more likely to complete TB treatment [7], although these results were mostly found in wealthy nation settings.

Technology would play a crucial role in achieving the WHO’s objective of eradicating TB by 2035 [10]. Advanced diagnostic tools, treatment monitoring systems, data management solutions, telemedicine, and research innovations, including surveillance, case management, contact tracing, and TB/LTBI screening, are crucial elements in the fight against TB. Harnessing the power of technology can improve TB detection, treatment outcomes, data-driven decision-making, and healthcare access, ultimately contributing to the goal of TB elimination. In achieving the objective of WHO, telemedicine could be at the forefront of leading the battle against TB.

In the context of this study, and according to the WHO, telemedicine refers to the provision of health services by health professionals, where distance is a critical factor, using information and communication technologies to exchange valid information for the purposes of diagnosis, treatment, and prevention of disease and injury, research and evaluation, and to facilitate the continuing education of health professionals, with the aim of safeguarding the health of individuals and communities. Telemedicine holds great promise for the treatment of TB [11]. It offers opportunities for remote consultations and expert advice, particularly in areas with limited access to specialized healthcare providers. Through videoconferencing and teleconsultation platforms, healthcare professionals can extend their expertise, support diagnosis, and guide TB management [12]. Telemedicine can help address the shortage of skilled healthcare workers and improve access to quality TB care in underserved regions.

Telemedicine allows healthcare providers to conduct remote consultations with TB patients, enabling access to specialized care even in areas with limited healthcare resources [13]. This approach is effective in improving patient outcomes and reducing the burden on healthcare facilities. By leveraging videoconferencing and digital platforms, telemedicine facilitates timely diagnosis, treatment monitoring, and adherence support for TB patients. Directly observed treatment (DOT) traditionally involves in-person observation of medication intake. However, telemedicine enables remote DOT, allowing healthcare providers to visually verify patients’ medication adherence through video calls, which serve as a direct measure of adherence. Additionally, electronic pillboxes, an indirect adherence monitoring tool, can track medication usage by recording when the pillbox is opened [14]. Remote DOT, whether through direct video observation or indirect electronic monitoring, has shown promising results in improving treatment adherence and reducing costs associated with in-person visits. Through teleconsultation, healthcare professionals can discuss complex cases, receive recommendations for treatment adjustments, and access up-to-date medical knowledge [15]. This approach improves patient convenience, reduces healthcare costs, and allows for efficient allocation of resources [16].

DiStefano and Schmidt [17] presented a framework to support the ethical planning, implementation, and evaluation of mobile health (mHealth) interventions for tuberculosis (TB) treatment adherence. They emphasized the potential of mHealth interventions, including mobile phone-based technologies, in improving TB treatment adherence. However, they also highlighted the importance of considering ethical implications throughout the development and implementation process. This review, therefore, aims to scope the existing literature on the adherence and treatment outcomes of tuberculosis patients treated through telemedicine.

## 2. Methodology

### 2.1. Literature Search

A systematic literature search was conducted to assess the impact of telemedicine-based directly observed therapy (DOT) on treatment adherence and outcomes in both pulmonary tuberculosis (PTB) and latent tuberculosis infection (LTBI) patients. The review focused on telecommunication technologies such as video directly observed therapy (VDOT), text messaging, and electronic reminders.

### 2.2. Search Strategy and Selection Criteria

The search was conducted following the Preferred Reporting Items for Systematic Reviews and Meta-Analyses (PRISMA) guidelines [18]. Four electronic databases—PubMed, Google Scholar, EMBASE, and Cochrane Library—were used to identify relevant studies published between 2010 and 2023.

The search terms included a combination of Medical Subject Headings (MeSH) and free-text keywords, such as “tuberculosis”, “pulmonary tuberculosis”, “latent tuberculosis”, “treatment adherence”, “medication adherence”, “treatment outcome”, “telemedicine”, “directly observed therapy”, “DOT” and “video DOT (VDOT)”. These terms were used individually and in combination to maximize the retrieval of relevant studies.

#### 2.2.1. Inclusion Criteria

Studies were included if they were published between 2010 and 2023 and were full-text, peer-reviewed articles. Only studies assessing the impact of telemedicine on treatment adherence or outcomes in pulmonary tuberculosis (PTB) or latent tuberculosis infection (LTBI) patients were considered. Eligible studies included observational studies, randomized controlled trials (RCTs), or systematic reviews that examined telemedicine interventions for TB treatment. Additionally, studies had to be published in English and involve human subjects.

#### 2.2.2. Exclusion Criteria

Studies were excluded if only abstracts were available, as full-text access was necessary for quality assessment. Articles outside the scope of PTB and LTBI treatment adherence or outcomes were removed. Studies that did not focus specifically on telemedicine interventions for TB treatment were also excluded. Duplicate studies and conference papers lacking sufficient data were eliminated. Additionally, any pilot studies published before 2010 were excluded to maintain consistency with the stated selection criteria.

### 2.3. Data Extraction, Critical Appraisal, and Synthesis

A total of 14,500 articles were retrieved from the four selected databases. After duplicate removal and an initial screening based on titles and abstracts, 374 articles remained. Two independent reviewers conducted a full-text assessment, applying the predefined inclusion and exclusion criteria. Following this rigorous screening, 14 studies were ultimately included in the review.

For each included study, data were extracted under key categories such as author(s), year of publication, study location, sample size, type of telemedicine intervention used (e.g., VDOT, SMS reminders), and primary findings related to treatment adherence and outcomes. The final selection of studies was based on their methodological quality and relevance to the research question, ensuring a comprehensive and unbiased assessment of telemedicine’s role in improving TB treatment adherence.

## 3. Results

**Figure 1:**
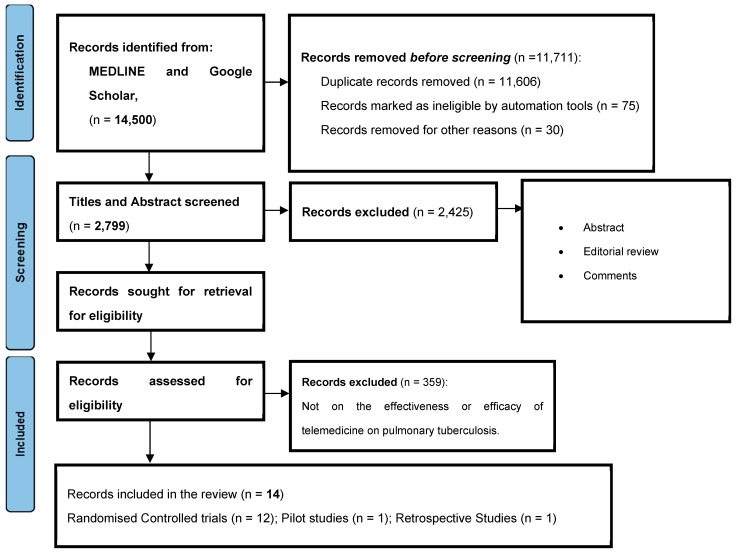
The PRISMA flowchart of the studies selected for review.

The studies highlighted here, as shown in Table 1, were reviewed for the effectiveness of telemedicine in the treatment of pulmonary tuberculosis. There were 12 randomized controlled trial studies, 2 pilot studies, and only 1 retrospective study as shown in Figure 1. Six studies utilized video directly observed therapy (VDOT), 3 studies used text message reminders (TMRs), while the remaining studies used other telemedicine-based interventions, including combined VDOT and TMRs, TMRs, and voice calls as reminders to monitor patients’ adherence.

The study conducted by Garfein et al. [19] showed that among the 27 patients who completed the study, 96% of observed therapy sessions were recorded and successfully transmitted. Burzynski et al. [20] demonstrated that electronic DOT was non-inferior to in-person DOT in terms of the percentage of completed doses. The study conducted by DeMaio et al. [21] demonstrated that telemedicine has the potential to enhance TB care by improving treatment adherence and patient satisfaction while reducing healthcare costs and logistical challenges associated with in-person observation.

Guo et al. [22] found high rates of treatment completion in both the VDOT and DOT groups, with no statistical differences between the two methods. Despite that, VDOT was associated with a significantly shorter time per observed dose compared to DOT, as well as lower costs. In India, Holzman et al. [23] (2019) suggested that VDOT is a feasible and acceptable method for TB treatment monitoring.

Manyasewal et al. [24] suggested that medication event reminder and monitor-observed therapy can improve health-related quality of life and reduce catastrophic costs in patients with TB compared to standard DOT. In a study conducted by Chuck et al. [25], treatment completion with VDOT was similar to that of in-person DOT, but adherence to scheduled VDOT sessions was better compared with in-person DOT. Lam et al. [26] observed that treatment adherence was found to be high for patients with latent tuberculosis infection on 3-month isoniazid and rifapentine, with participants reporting an average adherence rate of 96% across the treatment period. Browne et al. [27] did not record any significant difference in adherence between the wirelessly observed therapy and DOT groups, with 85.5% and 82.8% of participants completing their treatment, respectively. Clinical response and adverse events did not differ significantly between the groups.

Liu et al. [28] concluded in their study that electronic reminders, such as SMS text messages or phone calls, were effective in improving medication adherence and treatment completion rates among TB patients. Chen et al. [29] concluded that using synchronous video-observed treatment (SVOT) as a method for medication adherence in LTBI patients provided an advantage in privacy protection while improving treatment adherence and completion rates. Mohammed et al. [30] concluded in their study that implementing a daily SMS medication reminder system significantly improved treatment outcomes for patients with TB. The use of SMS reminders increased treatment success, adherence, and patient engagement, thereby contributing to better TB treatment outcomes through its cost-effectiveness.

Farooqi et al. [31] concluded that mobile SMS reminders can play a significant role in improving medication compliance among patients receiving anti-TB treatment through the DOTS program. The use of SMS reminders proved to be an effective and feasible intervention in promoting adherence to the prescribed medication regimen, leading to higher treatment completion rates and improved treatment outcomes in TB patients. Guo et al. [32] (2020) concluded that the comprehensive app utilizing video-observed therapy for TB treatment management was well-received by the participants and demonstrated good usability. Belknap et al.’s [33] study concluded that self-administered once-weekly isoniazid and rifapentine treatment for LTBI was superior to DOT in terms of treatment completion. Johnston et al. [34] demonstrated that text message reminders significantly improved adherence to treatment for LTBI.

**Table 1:** Summary of the treatment outcomes of pulmonary tuberculosis patients treated with telemedicine.

SN	Author/ Year/Country	Title	Goal/Objective	Methodology	Sample Size	Intervention	Result/Findings
1.	Lam et al. [26] (2018) USA	“Using video technology to increase treatment completion for patients with latent tuberculosis infection on 3-month isoniazid and rifapentine: an implementation study”	Assessing the use of video technology on improving completion of treatment in patients undergoing a 3-month rifapentine and isoniazid regimen for latent tuberculosis infection (LTBI).	Randomized controlled trial	116	Scheduled VDOT session	Use of VDOT significantly improved treatment completion rates compared to historical controls.
2.	Burzvnski et al. [20] (2022) USA	“In-person vs. electronic directly observed therapy for tuberculosis treatment Adherence: A Randomized Noninferiority”	To determine if electronic directly observed therapy (DOT) for monitoring tuberculosis treatment can achieve a comparable treatment level to in-person DOT.	Randomized crossover	216	Monitoring treatment of tuberculosis with electronic directly observed therapy (DOT)	This trial demonstrated that electronic DOT was non-inferior to in-person DOT in terms of the percentage of completed doses.
4.	Garfein et al. [35] (2018) USA	“Tuberculosis treatment monitoring by video directly observed therapy in 5 health districts, California, USA”	Examines the implementation and effectiveness of video directly observed therapy (VDOT) in five health districts in California, USA for monitoring tuberculosis (TB) treatment.	Retrospective study	467	Video directly observed therapy (VDOT)	VDOT was particularly effective in improving treatment adherence among specific patient groups, including those with substance use disorders, homeless individuals, and patients with previous treatment non-adherence.
5.	Guo et al. [32] (2020)	“A comprehensive app that improves tuberculosis treatment management through video-observed therapy: usability study”	To evaluate the cost and clinical benefits of video directly observed therapy (VDOT) in comparison to traditional directly observed therapy (DOT) for tuberculosis (TB) treatment.	Randomized controlled trial	405	Video directly observed therapy (VDOT)	High rates of treatment completion in both the VDOT and DOT groups, with no statistical differences between the two methods. VDOT was associated with a significantly shorter time per observed dose compared to DOT, as well as lower costs. Patients in the VDOT group reported better experiences and higher levels of satisfaction compared to those in the DOT group. They found VDOT to be convenient and comfortable and expressed a willingness to recommend the method to other patients.
6.	Holzman et al. [23] (2019) India	“Use of smartphone-based video directly observed therapy (VDOT) in tuberculosis care: a single-arm, prospective feasibility study”	To assess its feasibility and acceptability for TB treatment monitoring.	Pilot study	25	Video directly observed therapy (VDOT)	More than 90% of patients find it easy to make and upload videos. These findings suggest that vDOT is an acceptable and feasible method for the monitoring of TB treatment in India, expanding the evidence base for VDOT in resource-limited settings and first documenting the use of VDOT in India.
7.	Manyasewal et al. [24] (2022) Ethiopia	“Effect of digital medication event reminder and monitor-observed therapy vs. standard directly observed therapy on health-related quality of life and catastrophic costs in patients with tuberculosis: A secondary analysis of a randomized clinical Trial”	Investigating the impact of a digital medication event reminder monitor (MERM)-observed therapy compared to traditional directly observed therapy (DOT) on catastrophic costs and health-related quality of life (HRQoL) in patients with tuberculosis (TB) in a resource-constrained setting.	Randomized clinical trial	109	Digital medication event reminder monitor (MERM)	The median index value for EQ-5D-5L and HRQoL was significantly higher in the MERM-observed therapy group compared to the control group. Additionally, the intervention group had significantly lower median costs compared to the control group, resulting in potential cost savings.
8.	Chuck et al. [25] (2016) USA	“Enhancing management of tuberculosis treatment with video directly observed therapy in New York City”	To evaluate the effectiveness of video directly observed therapy (VDOT) in improving treatment outcomes for tuberculosis (TB) patients in New York City.	Randomized controlled trial	201	Video directly observed therapy (VDOT)	High levels of satisfaction were reported by patients in the VDOT group with the technology, and VDOT was found to be convenient and user-friendly. Healthcare providers also reported positive experiences with the VDOT system, finding it to be an effective tool for monitoring patients’ medication adherence.
9.	Browne et al. [27] (2019)	“Wirelessly observed therapy compared to directly observed therapy to confirm and support tuberculosis treatment adherence”	To compare wirelessly observed therapy (WOT) with directly observed therapy (DOT) in confirming and supporting adherence to tuberculosis (TB) treatment.	Randomized controlled trial	175	Smartphone that was equipped with a video-based adherence system	Both WOT and DOT groups had high levels of treatment adherence, with mean adherence rates of 90.7% and 90.6%, respectively. There was no significant difference in adherence between the two groups. Treatment completion rates were also similar between the WOT and DOT groups, with 85.5% and 82.8% of participants completing their treatment, respectively. Clinical response and adverse events did not differ significantly between the groups.
10.	Liu et al. [28] (2015) China	“Effectiveness of electronic reminders to improve medication adherence in tuberculosis patients”	To estimate the effectiveness of electronic reminders in improving medication adherence among tuberculosis (TB) patients.	Randomized controlled trial	4173	Patients received electronic reminders in the form of phone calls or texts in short message service (SMS)	The intervention group, receiving electronic reminders, had a higher significance in treatment completion rate when compared to the normal control.
11.	Chen et al. [29] (2020)	“Advantage in privacy protection by using synchronous video-observed treatment enhances treatment adherence among patients with latent tuberculosis infection”	To investigate the impact of synchronous video-observed treatment (SVOT) on adherence to treatment among latent tuberculosis infection (LTBI) patients.	Randomized controlled trial	200	Video call application to connect with healthcare providers	The SVOT group had significantly higher treatment adherence (98.2%) compared to the SAT group (89.0%). Furthermore, group SVOT had a higher treatment completion rate (92.0%) compared to the SAT group (81.0%). Patient satisfaction was also higher in the SVOT group, with 96.0% of participants expressing satisfaction with the SVOT intervention.
12.	Mohammed et al. [30] (2016)	“Impact of a daily SMS medication reminder system on tuberculosis treatment outcomes”	To assess how effective a daily SMS medication reminder would improve treatment outcomes in tuberculosis (TB) patients.	Randomized controlled trial	200	Daily SMS reminders	The intervention group had higher significance in treatment success (92.0%) and treatment adherence, with 94.0% of participants reporting good adherence when compared to patients in the control group, with 78.0% and 79.0%, respectively. Additionally, the intervention group had a lower treatment failure rate and a lower loss to follow-up rate compared to the control group.
13.	Farooqi et al. [31] (2017)	“The role of mobile SMS reminders in improving drug compliance in patients receiving anti-TB treatment from DOTS program”	To assess the effectiveness of mobile SMS reminders in improving medication compliance among patients receiving anti-tuberculosis (TB) treatment through the directly observed treatment short-course (DOTS) program.	Randomized controlled trial	300	Mobile SMS reminders	The intervention group had significantly higher medication compliance when compared to patients in the control group. A lower proportion of missed doses was observed in the intervention group, indicating better adherence to the regime of prescribed medication. Furthermore, a higher treatment success rate and treatment completion rate were recorded in the intervention compared to the control group. Patient satisfaction with the SMS reminder system was also reported to be high.
14.	Johnston et al. [34] (2018)	“The effect of text messaging on latent tuberculosis treatment adherence: a randomized controlled trial”	To investigate the influence of text messaging on adherence to treatment for latent tuberculosis infection (LTBI).	Randomized controlled trial	133	Text message reminders	The group that received text messages had significantly higher adherence to LTBI treatment compared to the standard care group. Adherence rates were 87.5% in the text message group and 75.0% in the standard care group. The text message intervention also resulted in a higher treatment completion rate (92.3% vs. 81.3%) and greater participant satisfaction. When the occurrence of adverse events between the two groups was observed, there were no significant differences, indicating that text messaging did not pose any additional risks.
15.	Guo et al. [22] 2020	Telemedicine technologies and tuberculosis management: a randomized controlled trial	To assess the clinical and cost-benefit of video directly observed therapy (VDOT), compared with DOT service.	Randomized controlled trial	405 participants from each study arm	Video directly observed therapy (VDOT)	VDOT enabled meaningful direct observation for TB patients through mobile devices, which was highly acceptable to patients and health care providers. It also saves time and is a cost-effective method, enabling the use of the saved money in other much-needed areas for TB.

## 4. Discussion

This review evaluated 15 studies published between 2010 and 2023 that examined the impact of telemedicine-based directly observed therapy (DOT) on tuberculosis (TB) treatment adherence and outcomes. The studies included various methodological designs, including 12 randomized controlled trials (RCTs), two pilot studies, and one retrospective study. Most of the research was conducted in high-income countries, with a limited number of studies from resource-limited settings such as Africa and Asia, where TB prevalence is highest.

There was considerable heterogeneity across the studies in terms of study design, intervention methods, and adherence measurement. Some studies assessed adherence based on medication ingestion rates, while others measured adherence by the percentage of completed doses. The use of video directly observed therapy (VDOT), text message reminders (TMRs), and electronic DOT varied across the studies, with some studies combining multiple interventions. This variability in adherence definitions makes direct comparisons difficult and highlights the need for standardized adherence metrics in future research. Establishing universally accepted metrics for adherence, such as validated electronic monitoring systems or biochemical verification, would enhance comparability and reproducibility across studies.

Most studies included in this review were conducted in high-income countries, particularly the United States. However, TB prevalence is highest in low- and middle-income countries, particularly in Africa and Asia, where access to healthcare and adherence monitoring remains a significant challenge. Limited research from these regions suggests an urgent need for more studies evaluating the effectiveness of telemedicine interventions in resource-limited settings. Future research should focus on the feasibility, scalability, and cost-effectiveness of telemedicine solutions in these regions, particularly where healthcare infrastructure is weak, and patient follow-up remains challenging.

While many studies focused on treatment completion rates, several also reported high levels of patient satisfaction and cost savings associated with telemedicine interventions. Studies such as those by DeMaio et al. [21], Guo et al. [22], Chuck et al. [25], and Holzman et al. [23] demonstrated that VDOT was perceived as convenient and effective by both patients and healthcare providers. Patients reported increased comfort, privacy, and flexibility when using remote monitoring systems. Manyasewal et al. [24] and Mohammed et al. [30] also highlighted the cost-effectiveness of telemedicine, as it reduced travel costs and healthcare expenses for both patients and healthcare facilities. These findings indicate that beyond improving adherence, telemedicine has the potential to enhance the overall patient experience and reduce financial burdens associated with TB treatment.

The methodological diversity among the reviewed studies influenced the reported outcomes. RCTs, such as those by Burzynski et al. [20] and Lam et al. [26], provided strong evidence supporting the effectiveness of VDOT, while pilot studies, such as Holzman et al. [23], primarily demonstrated feasibility rather than long-term adherence impact. Retrospective studies, such as Garfein et al. [35], offered insights into real-world applications of telemedicine interventions but lacked the rigorous control of RCTs. The differences in methodology highlight the need for well-designed, large-scale RCTs in diverse settings to provide more conclusive evidence on the efficacy of telemedicine DOT in TB treatment.

To maximize the potential benefits of telemedicine in TB management, several key strategies should be implemented in resource-limited regions. Infrastructure development is essential, requiring investment in stable internet access, mobile technology, and digital health platforms to ensure the scalability of telemedicine interventions. Training and capacity building must be prioritized so that healthcare providers and patients are well-equipped to use telemedicine tools effectively. Additionally, integration with existing TB programs should be emphasized, allowing telemedicine solutions to complement traditional directly observed therapy (DOT) within national TB control efforts to enhance sustainability [36]. A patient-centered approach is crucial, addressing concerns about privacy, usability, and cultural acceptability to improve patient engagement and adherence. Finally, affordability and accessibility should be ensured by exploring subsidized digital health services or fostering partnerships with mobile network providers to reduce costs for patients, making telemedicine a viable solution for TB treatment in low-resource settings.

Future studies should prioritize evaluating telemedicine DOT in high-TB-burden regions, particularly in Africa and Asia, to determine its feasibility and impact in these settings [37]. Additionally, research should explore the effectiveness of hybrid approaches that combine telemedicine with community-based interventions to address adherence barriers. Standardizing adherence measurement tools and conducting cost-effectiveness analyses tailored to different healthcare systems will further enhance the implementation and scalability of telemedicine for TB treatment.

## 5. Conclusions

The evidence synthesized from the reviewed studies underscores the effectiveness of telemedicine in improving treatment adherence and outcomes among patients with pulmonary tuberculosis (PTB). The diverse interventions, ranging from video directly observed therapy (VDOT) to text message reminders (TMRs), have demonstrated promising results in enhancing medication adherence, facilitating real-time monitoring, and providing personalized care for TB patients. Despite variations in study designs, settings, and populations, the consistent findings point toward the potential of telemedicine to address the challenges of patient non-adherence and treatment discontinuation, which have long been barriers to effective TB management. Moreover, telemedicine offers a convenient, accessible, and cost-effective approach to supporting patients in remote or underserved areas, bridging gaps in healthcare access and delivery.

However, it is important to acknowledge the limitations and gaps in the existing literature, including the predominance of studies conducted in developed nations and the scarcity of robust trials in resource-constrained settings, particularly in Africa. Future research should aim to address these disparities and generate more evidence on the scalability, feasibility, and sustainability of telemedicine interventions in diverse healthcare contexts. Additionally, investments in healthcare infrastructure, internet connectivity, and digital literacy are essential to enable widespread telemedicine adoption and maximize its potential impact on TB control efforts.

Overall, telemedicine represents a promising tool in the fight against tuberculosis, offering innovative solutions to enhance treatment adherence, improve patient outcomes, and ultimately contribute to the global goal of TB elimination by 2035. Through continued research, collaboration, and investment, telemedicine can play a pivotal role in shaping the future of TB care delivery and advancing toward a TB-free world.

## 6. Limitations of the Review

While the strength of the review lies in its comprehensive examination of the existing literature on telemedicine’s application in tuberculosis (TB) treatment, providing valuable insights into the effectiveness of various telemedicine interventions, the review also has some limitations that warrant consideration. Understanding and addressing these limitations are crucial for interpreting the findings accurately and guiding future research efforts aimed at optimizing telemedicine strategies for TB management.

1. Geographical Bias: The majority of the studies included in this review were conducted in developed nations, leading to potential geographical bias. Limited representation from resource-constrained settings, particularly in Africa and other low-income regions, may restrict the generalizability of findings to diverse healthcare contexts.
2. Study Design Variability: The studies encompassed a variety of designs, including randomized controlled trials (RCTs), pilot studies, and retrospective analyses. While each design offers unique insights, the variability in methodologies makes it challenging to directly compare findings and draw definitive conclusions.
3. Population Heterogeneity: The patient populations across the reviewed studies exhibited considerable heterogeneity in terms of demographic characteristics, TB severity, comorbidities, and socioeconomic status. This heterogeneity may introduce confounding variables that could influence treatment outcomes and adherence rates.
4. Intervention Diversity: The telemedicine interventions evaluated in the studies varied widely, encompassing video directly observed therapy (VDOT), text message reminders (TMRs), and other telecommunication technologies. While this diversity reflects the evolving landscape of telemedicine, it also complicates the synthesis of results and limits the ability to identify optimal intervention strategies.
5. Limited Longitudinal Data: Many of the reviewed studies provided short-term outcomes, such as treatment completion rates and adherence metrics. Longitudinal data on sustained treatment outcomes, relapse rates, and long-term patient follow-up were often lacking, limiting the assessment of telemedicine’s durability and effectiveness over time.

## 7. Recommendations

- Clear guidelines and protocols need to be developed and implemented to ensure standardized and effective telemedicine practices in TB treatment. These guidelines should address issues such as patient selection criteria for telemedicine, data privacy, and security measures, as well as ethical considerations specific to telemedicine practice.
- Healthcare professionals, including nurses, should receive comprehensive training on telemedicine technologies, platforms, and best practices. Continued professional development opportunities and training programs should also be provided to ensure healthcare professionals remain updated on evolving telemedicine practices.
- Expanding internet access and ensuring reliable connectivity in remote regions will enable effective telemedicine implementation and bridge the digital divide, ensuring equal access to TB care, most especially in developing countries.
- Further research is needed to evaluate the long-term impact and effectiveness of telemedicine in TB treatment. Studies should focus on outcomes such as treatment adherence, treatment success rates, cost-effectiveness, patient satisfaction, and healthcare provider experiences. The findings from such studies will provide valuable insights and evidence to guide future telemedicine interventions and inform policy decisions.
- Collaboration among healthcare institutions, professional associations, and researchers is crucial for sharing best practices and experiences related to telemedicine in TB treatment.
- Efforts should be made to ensure equitable access to telemedicine services for all TB patients with special attention to vulnerable populations, such as those in rural or underserved areas, individuals with low socioeconomic status, and marginalized communities. Strategies like subsidized internet access or mobile data plans can help address access barriers and ensure inclusivity.
